# Anti-Obesity Effects of Dietary Fibers Extracted from Flaxseed Cake in Diet-Induced Obese Mice

**DOI:** 10.3390/nu15071718

**Published:** 2023-03-31

**Authors:** Manman Zhao, Beibei Wang, Li Li, Wei Zhao

**Affiliations:** State Key Laboratory of Food Science and Technology, School of Food Science and Technology, Jiangnan University, Wuxi 214122, Chinalili.zz@jiangnan.edu.cn (L.L.)

**Keywords:** flaxseed cake, insoluble dietary fiber, soluble dietary fiber, high-fat diet, lipid accumulation, gut microbiota

## Abstract

Although many efforts have been made to characterize the functional properties of flaxseed, knowledge concerning the properties of insoluble and soluble dietary fibers in flaxseed is still limited. Here, insoluble and soluble dietary fibers were extracted from flaxseed cake—a valuable resource that has not been fully exploited. Subsequently, their monosaccharide compositions, structural properties, and anti-obesity effects in male mice were characterized. The anti-obesity effects of flaxseed cake insoluble dietary fiber (FIDF), flaxseed cake soluble dietary fiber (FSDF), and FIDF combined with FSDF in diet-induced obese mice were investigated in our study. Supplementation with FSDF alone or FIDF and FSDF together lowered the fat accumulation, improved the serum lipid profile, increased the basal metabolism, and improved the gut microbiota of obese mice. Supplementation with FIDF and FSDF together significantly enriched the abundance of *g_Akkermansia* and *g_Bifidobacterium*, which are negatively associated with obesity. Supplementation with FIDF alone improved the liver lipid profile, raised the basal metabolism, and enhanced the short-chain fatty acid levels in the guts of the mice. In conclusion, our results collectively support the therapeutic potential of FIDF and FSDF in obesity treatment and indicate that FIDF and FSDF play different roles in the process of obesity treatment. Furthermore, our results provide critical information for flaxseed cake resource exploitation.

## 1. Introduction

In recent years, the rapid rise in obesity worldwide has led to its evolution into a global epidemic and a major health challenge. As a chronic, recurrent, and progressive disease, obesity is a major contributor to numerous comorbidities, including metabolic syndrome and cardiovascular diseases [1]. A series of epidemiological and intervention studies have shown that high-fiber diets can reduce the risk of obesity and its associated comorbidities [2,3,4]. Consumers’ interest in high-fiber diets has been increasing due to their beneficial effects, and a daily intake of 25 g to 29 g of dietary fiber is recommended [5]. Dietary fiber (DF) is a mixture of polysaccharides and complex sugar chain structures [6], and its health benefits can be influenced by many factors, e.g., the source and type of DF. Flaxseed (*Linum usitatissimum*) cake is a by-product of flaxseed following oil extraction, and it is a valuable resource for dietary fibers, though it has not been fully commercially exploited [7]. The dietary fiber content of flaxseed cake is about 35–45% [8], including insoluble dietary fiber (mainly in the flaxseed hull) and water-soluble heterogeneous polysaccharides (soluble dietary fiber) [9]. The health benefits of the soluble and insoluble dietary fibers extracted from flaxseed cake are still unknown.

Flaxseed has been approved as a common food raw material due to its various potential health benefits [10]. Flaxseed cake retains most of the nutrients of flaxseed and it can be used in the production of functional food, although this is still in the early stages of exploration [11,12]. Yu et al. have reported that expanded flaxseed cake could effectively regulate blood glucose and lipid metabolism in rats on a high-fat and high-sugar diet [13]. A controlled crossover trial in human beings showed that the intervention of partially defatted flaxseed cake could reduce total cholesterol by 4.6% and low-density lipoprotein cholesterol by 7.6% [14]. As the main component of flaxseed cake, the dietary fibers’ role in regulating lipid metabolism is still not clear [15]. Dietary fibers obtained from different sources vary in composition, structure, solubility, and so on, resulting in different degrees of fermentation in the large intestine and different metabolic effects [16,17]. Different types of dietary fiber have different physical and chemical properties, determining whether they exhibit beneficial effects through different pathways [18]. Previous studies have indicated that, compared with IDF, the SDF extracted from bergamot adsorbs cholesterol and glucose more effectively due to its porous structure [19]. IDF plays a beneficial role in promoting defecation [20] and supporting the growth of intestinal microorganisms [21]. Thus, it is worth evaluating and comparing the health benefits of flaxseed cake insoluble dietary fiber (FIDF) and flaxseed cake soluble dietary fiber (FSDF).

In the present study, flaxseed cake was enzymatically hydrolyzed to obtain FIDF and FSDF fractions, and their monosaccharide compositions, structural properties, and anti-obesity properties in vivo were characterized. The interventions with FIDF or FSDF alone and the intervention with FIDF and FSDF together were performed on male C57BL/6J obese mice, and the effects of soluble and insoluble DFs on obesity treatment were then investigated in terms of body weight, lipid metabolic capacity, energy metabolism, gut microbiota, and so on. This is expected to provide theoretical bases for the further comprehensive development and utilization of flaxseed cake resources and to offer useful information regarding how to effectively use dietary fiber supplementation to improve human health.

## 2. Materials and Methods

### 2.1. Materials

Flaxseed cake was purchased from Xinjiang Yangreen Agricultural Technology Co., Ltd. (Alashankou, Xinjiang, China). Heat-stable α-amylase and alkaline protease were purchased from Novozymes Biotechnology Co., Ltd. (Tianjin, China). Standards of monosaccharides were purchased from Aladdin Chemistry Co., Ltd. (Shanghai, China). All other chemicals and reagents used were analytical grade and commercially available.

### 2.2. Preparation of FIDF and FSDF

Pieces of flaxseed cake were ground finely into 40-mesh powder. FIDF and FSDF were extracted from flaxseed cake powder via enzymatic hydrolysis using the AOAC method 991.43 with a little modification. First, 20 g of dried flaxseed cake powder was dissolved in deionized water (*w*/*v*, 1:20), and the solution was treated with α-amylase at 95 °C for 35 min (enzyme dosage 0.3%). The pH was then adjusted to 9.5 ± 0.2 at 60 °C, alkaline protease was added (enzyme dosage 0.6%), and the mixture was incubated at 65 °C for 4 h. After the enzymatic hydrolysis, the mixture was centrifuged at 7020× *g* (GL-21 MS high-speed centrifuge, Shanghai Luxiangyi Centrifuge Instrument Co., Ltd., Shanghai, China) at 4 °C for 5 min with fixed-angle rotor model No. 10. The supernatant was then collected, and four volumes of 95% (*v*/*v*) ethanol were added to precipitate the SDF for 12 h. Next, the precipitate was dried to obtain the flaxseed cake soluble dietary fiber. The residue after centrifugation was washed and centrifuged twice, and then dried to obtain the flaxseed cake insoluble dietary fiber. Both the FIDF and FSDF were crushed and sieved through the 60-mesh sieve. The purities of the flaxseed-extracted FIDF and FSDF were 78.6% and 70.1%, respectively.

### 2.3. Monosaccharide Composition Analysis

The monosaccharide composition was measured according to our previous method [22]. First, 5 mg of FIDF or FSDF was dissolved in 5 mL trifluoroacetic acid solution (2 M) and hydrolyzed at 121 °C for 2 h using a LDZX-50KBS vertical heating steam pressure sterilizer (Shanghai Shenan Medical Instrument, Shanghai, China). The hydrolysate was then dried using nitrogen blowing to remove any trifluoroacetic acid and dissolved in distilled water. Standards of the monosaccharides, including arabinose, fucose, rhamnose, galactose, glucose, xylose, mannose, fructose, glucuronic acid, and galacturonic acid were prepared. Each sample was analyzed using a DIONEX ICS-5000 ion chromatographic instrument (Thermo Fisher Scientific, Waltham, MA, USA).

### 2.4. Scanning Electron Microscopy (SEM) Analysis

The FIDF and FSDF powder samples were sputtered with gold under reduced pressure conditions. The morphological structures of the DFs were observed using a SU8100 cold field emission SEM (FEI Co., Hillsboro, OR, USA) at an acceleration voltage of 20 kV and a magnification coefficient of 10 k and 5 k.

### 2.5. Animal Experiments

All animal experiments were performed following the National Guidelines for Ethical Review of Experimental Animal Welfare. Four-week-old healthy C57BL/6J male mice were purchased from GemPharmatech Co., Ltd. (Nanjing, China). The mice were maintained in a clean, environmentally controlled room (temperature: 23 ± 1 °C; humidity: 50 ± 5%) under a 12 h light–dark cycle. All mice had free access to water and food. After 2 weeks of acclimatization, the mice were fed ad libitum with a normal diet (control group, 10% heat from fat, XTControl50J) or a high-fat diet (60% heat from fat, XTHF60) for 9 weeks. Subsequently, the successful HFD-fed mice were randomly assigned to four experimental groups (*n* = 6 for each group). The mice were continually fed either a high-fat diet (HFD group), a modified HFD containing 6.46% FIDF (FIDF group), a modified HFD containing 6.46% FSDF (FSDF group), or a modified HFD containing 3.23% FIDF + 3.23% FSDF (MIX group) for 5 weeks, separately. All the experimental diets were obtained from Jiangsu Synergetic Pharmaceutical Bioengineering Co., Ltd. (Nanjing, China), and the compositions and heat ratios of the five experimental diets are shown in Table S1. We measured the food consumption and body weight of the mice at least once a week.

### 2.6. The Determination of the Basal Metabolism of the Mice

For whole-body metabolic monitoring, we used a Comprehensive Laboratory Animal Monitoring System (CLAMS) (Columbus Instruments, Columbus, OH, USA). The oxygen consumption (VO_2_), carbon dioxide production (VCO_2_), heat production, and ambulatory locomotor activity were assessed. During the last week of treatment, mice were randomly selected and individually subjected to the twelve-chamber CLAMS for 36 h (12 h for acclimatization and 24 h for data collection, 4 mice per group). The respiratory exchange ratio (RER) was calculated as VCO_2_/VO_2_, and ambulatory activity refers to the mice’s total breaking numbers of infrared laser beams.

### 2.7. Sample Collection

Fresh fecal pellets were collected three days before the mice were euthanized, and these were kept at −80 °C for further study. At the end of treatment, the mice were fasted overnight, weighed, and then euthanized using carbon dioxide anesthesia and cervical dislocation. The brown adipose tissue (BAT), epididymal adipose tissue (eWAT), inguinal subcutaneous adipose tissue (iWAT), and liver tissue were rapidly isolated and accurately weighed. Serum was obtained by blood centrifugation at 1327× *g* (Centrifuge 5427R; Eppendorf Corporate, Germany) at 4 °C for 15 min with fixed rotor model FA-45-24-11. All collected samples were preserved at −80 °C for further analysis.

### 2.8. Serum Biochemical Assays

The serum lipid profile, including total cholesterol (TC), triglycerides (TG), high-density lipoprotein cholesterol (HDL-c), low-density lipoprotein cholesterol (LDL-c), total bile acid (TBA), uric acid (UA), alanine aminotransferase (ALT), and aspartate aminotransferase (AST), were assayed using a Mindrayn BS-480 automatic biochemical analyzer.

### 2.9. Liver Lipid Profile Assays

The TC and TG levels of the liver were determined using a total cholesterol assay kit (A111-1-1) and a triglyceride assay kit (A110-1-1), respectively, both of which were purchased from Nanjing Jiancheng Bioengineering Institute (Nanjing, China). Liver tissues were mixed with anhydrous ethanol (*w*/*v*, 1:9), fragmented using a sample fast grinding machine (JXFSTPRP-32L, Jingxin Industrial Development Co., Ltd., Shanghai, China) for 5 min, and then centrifuged at 1327× *g* (Centrifuge 5427R) for 10 min at 4 °C to obtain the supernatant for analysis.

### 2.10. Hepatic Histomorphology Analysis

A part of the liver tissue fixed in 4% paraformaldehyde was embedded in paraffin and cut into 4 um sections for hematoxylin-eosin (HE) staining. After dehydration, sections were sealed with neutral gum for histomorphology analysis. All of the stained sections were observed under a light microscope (Nikon, Tokyo, Japan).

### 2.11. Gut Microbiota Analysis

The DNA extraction and 16S rRNA amplicon sequencing were conducted according to our previous method [23]. An E.Z.N.A. soil DNA kit (Omega Bio-tek, Norcross, GA, USA) was used to extract DNA from the mice feces. Primers 338F and 806R were used to amplify the V3-V4 hypervariable region of the extracted DNA samples. After purification and quantification, the purified amplicons were paired-end sequenced on the Illumina MiSeq PE300 platform (Illumina, San Diego, CA, USA) according to the standard protocols of HonSunBio Technology Co., Ltd. (Shanghai, China).

### 2.12. Short-Chain Fatty Acid (SCFA) Analysis in Cecal Contents

Firstly, 50 mg of freeze-dried cecal contents were suspended in 0.5 mL of saturated NaCl solution, and the suspensions were homogenized at 4 °C. Secondly, 40 μL of 10% (*v*/*v*) sulfuric acid solution was added and the solution was shaken for 30 s. Next, 1 mL of anhydrous ether was added quickly to extract the SCFAs in an ice bath, and the solution was then shaken at 4 °C for 30 s. The upper ether phase was obtained by centrifugation at 15,600× *g* (4 °C, 15 min; Centrifuge 5427R). Thirdly, the upper ether phase was mixed with 0.25 g of anhydrous sodium sulfate and left to stand for 15 min to remove water. The solution was then centrifuged at 15,600× *g* (4 °C, 15 min; Centrifuge 5427R) to remove the sodium sulfate. Finally, the obtained supernatants were analyzed via gas chromatography (GC-2010 Plus, Shimadzu, Kyoto, Japan) using a Rtx-Wax chromatographic column.

### 2.13. Statistical Analysis

All test data were analyzed using IBM SPSS Statistics 25.0 software (Armonk, NY, USA). Data were presented as mean ± standard error of the mean (SEM) with at least three replicates. One-way analysis of variance (ANOVA) with Tukey’s test was used for comparison among groups. The threshold for statistical significance was set at *p* < 0.05.

## 3. Results

### 3.1. The Monosaccharide Composition of FIDF and FSDF

To better understand the properties of the two extracted dietary fibers, the monosaccharide compositions of the FIDF and FSDF were analyzed (Table 1). The FIDF sample contained seven monosaccharides, including fucose, rhamnose, arabinose, galactose, glucose, xylose, and galacturonic acid, whereas the FSDF sample contained six of these monosaccharides and did not contain fucose. Glucose in cellulose and xylose in hemicellulose were the two most abundant primary sugars in both the FIDF and the FSDF, followed by arabinose in the FIDF sample and galactose in the FSDF sample. The arabinose content was similar in the FIDF and the FSDF.

### 3.2. The Microstructure of FIDF and FSDF

**Figure 1:**
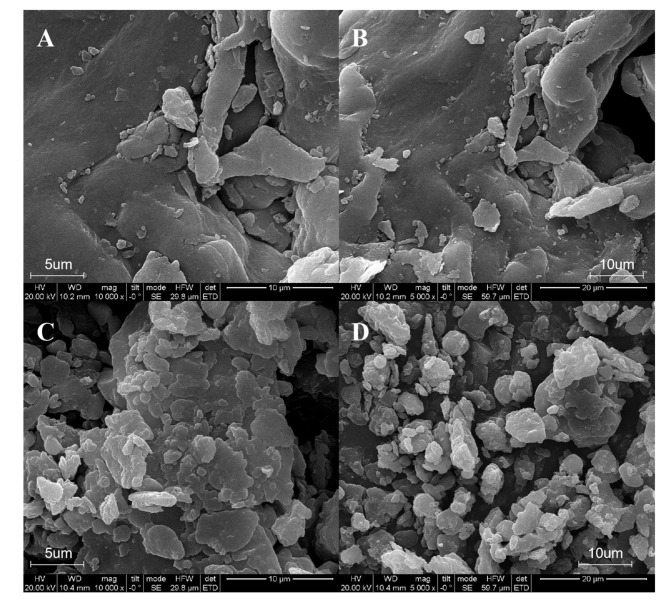
SEM analyses of FSDF and FIDF at 10,000× and 5000× magnification. (**A**) FSDF × 10,000; (**B**) FSDF × 5000; (**C**) FIDF × 10,000; (**D**) FIDF × 5000.

The SEM micrographs of the FIDF and FSDF under 10,000× and 5000× magnification are shown in Figure 1. The FSDF had an irregular and loose structure, with a partial sheet-like and partial strip-like appearance and an uneven surface (Figure 1A,B). The FIDF particles showed a porous coralloid structure and a more regular morphology than the FIDF particles (Figure 1C,D). The loose structure of the FSDF was supposed to increase its adsorption capacity, and the porous structural features of the FIDF were conducive to its binding capacity since it could immobilize the molecules in the holes [24,25].

### 3.3. Effects of FIDF and FSDF on Fat Accumulation and Serum Lipid Profiles

**Figure 2:**
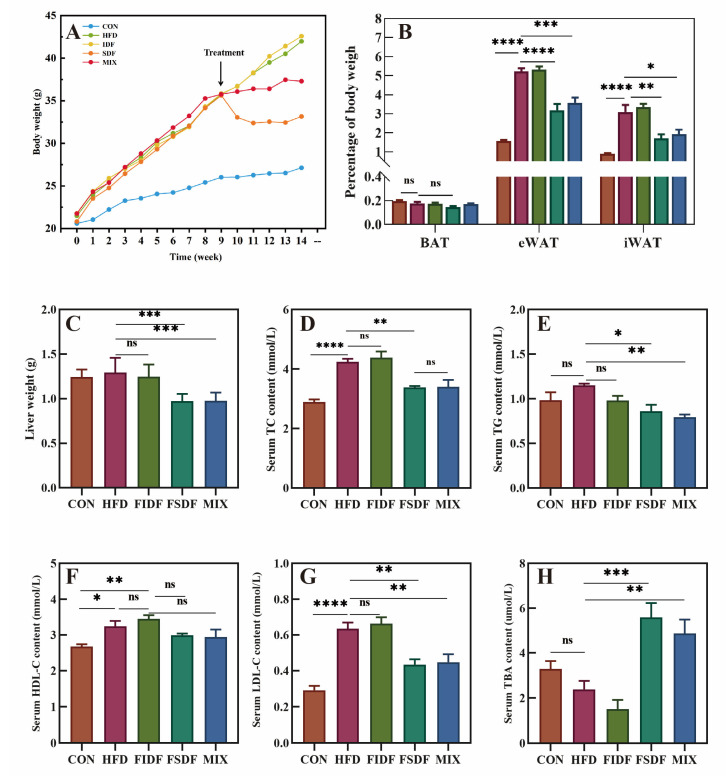
Effects of FIDF and FSDF on obesity symptoms and plasma biochemical parameters. (**A**) Body weight. (**B**) Percentage of BAT, eWAT, and iWAT to body weight. (**C**) Liver weight. (**D**–**H**) Serum TC, TG, HDL-C, LDL-C, and TBA contents, respectively. CON, control group; HFD, high-fat diet group; FIDF, group supplemented with FIDF alone; FSDF, group supplemented with FSDF alone; MIX, group supplemented with FIDF and FSDF together. Data are expressed as mean ± SEM (*n* = 6). * *p* < 0.05; ** *p* < 0.01; *** *p* < 0.0001; **** *p* < 0.0001; ns, not significant.

After we discovered the structural properties of the FIDF and FSDF, their anti-obesity effect was evaluated. After 9 weeks of feeding, the average weight of the mice fed a high-fat diet (35.42 ± 3.87 g) was more than 20% higher than that of the mice fed a normal diet (25.97 ± 0.58 g), revealing that the obese mice model was constructed successfully (Figure 2A). As is shown in Figure 2A, the FSDF or the mixture of FIDF and FSDF (MIX group) inhibited the body weight gain of the HFD mice after a one-week intervention and significantly reduced the mice’s body weight compared with the HFD mice after a five-week treatment. The mice in the FSDF and MIX groups also showed decreased body fat mass proportions and decreased liver weights compared with the HFD mice (Figure 2B,C). Notably, no significant difference in total energy intake was found between the treated and untreated obese mice during the five-week treatment, and the energy efficiency of the mice was lower in the FSDF and MIX groups than in the other groups (Table S2). The interventions with FSDF alone or FIDF and FSDF together appeared to play a protective role against the development of HFD-induced obesity in mice, while the intervention with FIDF alone had no significant effect on obesity development (Figure 2A–C). As is shown in Figure 2D–H, HFD feeding led to several significant alterations in serum metabolites, including increased levels of TC, HDL-c, and LDL-c. Both the FSDF and MIX mice exhibited distinct serum metabolite profiles, featuring reduced TC, TG, and LDL-c levels and elevated TBA levels compared with the untreated obese mice. Furthermore, all the obese mice, including the treated and untreated mice, showed approximate serum HDL-c (Figure 2F) and UA levels (Table S2).

**Figure 3:**
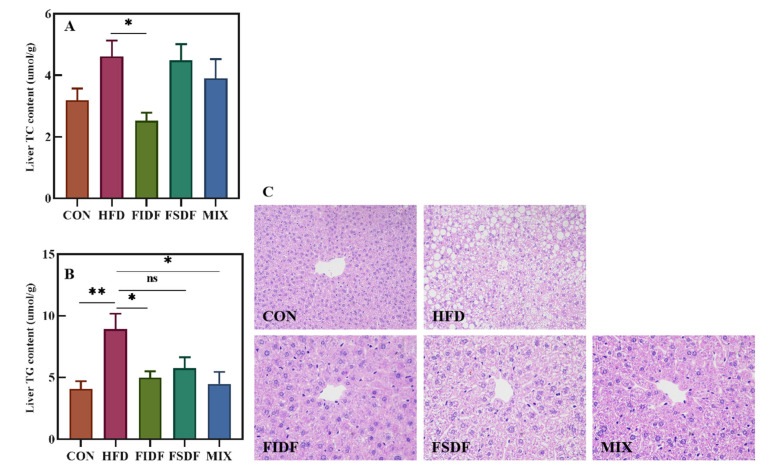
Effects of FIDF and FSDF on hepatic lipid accumulation and hepatic histomorphology. (**A**) Liver TC content. (**B**) Liver TG content. (**C**) HE staining of liver sections in different groups (shown at 400× magnification). CON, control group; HFD, high-fat diet group; FIDF, group supplemented with FIDF alone; FSDF, group supplemented with FSDF alone; MIX, group supplemented with FIDF and FSDF together. Data are expressed as mean ± SEM (*n* = 6). * *p* < 0.05; ** *p* < 0.01; ns, not significant.

Unlike the FSDF intervention, which improved serum lipid metabolism outstandingly well, the FIDF intervention displayed advantages in alleviating liver lipid accumulation (Figure 3). As is shown in Figure 3A,B, the FIDF intervention decreased the liver lipids in obese mice, showing a significantly lower level of hepatic TC and TG. In addition, the FIDF and FSDF interventions also significantly lowered the hepatic TG levels. The MIX mice had the lowest level of hepatic TG, followed by the FIDF mice and the FSDF mice. The FSDF intervention also decreased the hepatic TG levels, although with no statistical difference. The above results were consistent with the hepatic histomorphology results. As is shown in Figure 3C, obvious parenchymal steatosis and abnormal fat accumulation were observed in the hepatic cells of the HFD mice. The presence of extensive lipid droplets in various sizes and abnormally arranged swollen hepatocytes indicated the liver lesions induced by the HFD. All three dietary fiber treatments alleviated the liver damage, resulting in smaller-sized hepatocytes, fewer intracellular lipid droplets, and less parenchymal steatosis. It is worth noting that there were almost no apparent lipid droplets in the liver tissue of the FIDF mice.

### 3.4. Effects of FIDF and FSDF on Energy Expenditure, RER, and Physical Activity

**Figure 4:**
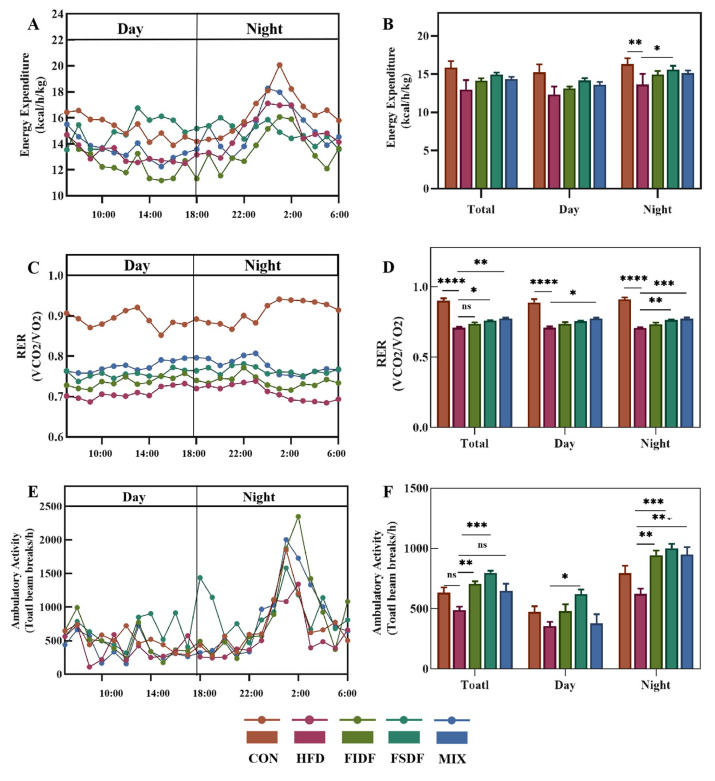
Effects of FIDF and FSDF on basal metabolism. The mice’s basal metabolism was measured by housing the mice in a twelve-chamber Comprehensive Laboratory Animal Monitoring System and using indirect calorimetry. (**A**) The 24 h light–dark cycle energy expenditure. (**B**) Total, light, and dark energy expenditure. (**C**) The 24 h light–dark cycle RER. (**D**) Total, light, and dark RER. (**E**) The 24 h light–dark cycle ambulatory activity. (**F**) Total, light, and dark ambulatory activity. CON, control group; HFD, high-fat diet group; FIDF, group supplemented with FIDF alone; FSDF, group supplemented with FSDF alone; MIX, group supplemented with FIDF and FSDF together. Data are expressed as mean ± SEM (*n* = 6). * *p* < 0.05; ** *p* < 0.01; *** *p* < 0.0001; **** *p* < 0.0001; ns, not significant.

Obesity is accompanied by energy imbalance due to increased energy intake and/or decreased energy expenditure. To further explore the mechanism of weight loss and improved lipid metabolism induced by the FIDF and FSDF, the whole-body basal metabolism was determined using indirect calorimetry during the last week of the treatments. As is shown in Figure 4A, the HFD-fed mice exhibited a decreased energy expenditure compared with their lean littermates (mice in the control group), and the FIDF and FSDF treatments weakened the effect of high-fat feeding to some extent. The energy expenditure of the FIDF mice (14.12 kcal/h/kg), the FSDF mice (14.92 kcal/h/kg), and the MIX mice (14.37 kcal/h/kg) increased by 8.8%, 15%, and 10.8%, respectively, compared with that of the HFD mice (12.97 kcal/h/kg) (Figure 4B). RER is an indicator of energy utilization, a RER value of 1 corresponding to pure carbohydrate oxidation and a RER value of 0.7 corresponding to pure lipid oxidation. The RER value of the control mice ranged from 0.85 to 0.95 during the 24 h light–dark cycle (Figure 4C), whereas it ranged from 0.68 to 0.74 for the HFD-fed mice (Figure 4C). These results were consistent with our expectations. The FIDF and FSDF treatments transferred the energy utilization of the mice from lipids to carbohydrates. Moreover, the MIX mice showed the highest RER level among the treated mice (Figure 4D). As is shown in Figure 4E,F, the HFD mice exhibited the least locomotor activity, and the FSDF mice exhibited a significant increase in ambulatory activity, both during the day and at night, compared with the HFD mice. The FIDF and MIX mice only significantly increased their ambulatory activity at night compared with the HFD mice. Thus, the FIDF and FSDF interventions mainly improved the mice’s locomotor activity at night. Our results suggest that the anti-obesity effect of the FIDF and FSDF may be achieved by increasing energy consumption and restoring energy balance.

### 3.5. Effects of FIDF and FSDF on Mice Gut Microbiota

**Figure 5:**
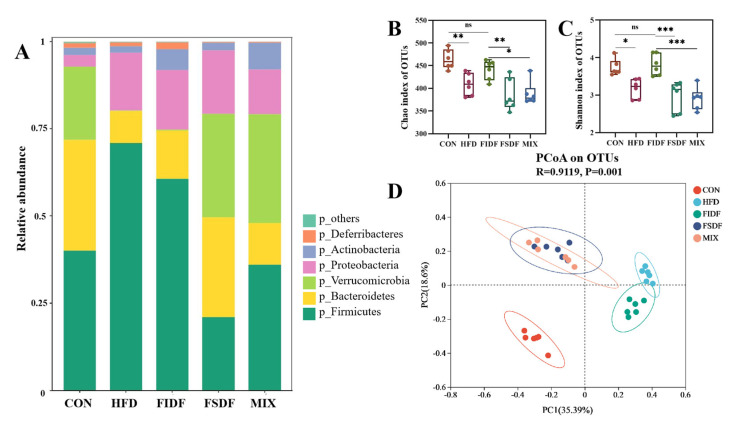
Effects of FIDF and FSDF on intestinal flora composition. (**A**) Microbial distribution at the phylum level. (**B**) Chao index at the OTU level. (**C**) Shannon index at the OTU level. (**D**) Principal coordinate analysis (PCoA) based on OTU levels. CON, control group; HFD, high-fat diet group; FIDF, group supplemented with FIDF alone; FSDF, group supplemented with FSDF alone; MIX, group supplemented with FIDF and FSDF together. Data are expressed as mean ± SEM (*n* = 6). * *p* < 0.05; ** *p* < 0.01; *** *p* < 0.0001; ns, not significant.

Growing evidence has proved the essential role of gut microbiota in the development of diet-induced obesity in mice. Thus, the bacterial communities in the fecal samples of the mice were analyzed. *Firmicutes* and *Bacteroidetes* were the dominant flora in all the samples, and *Verrucomicrobiota* accounted for a large proportion in mice from the control, FSDF, and MIX groups (Figure 5A). Compared with the HFD group, the FIDF and FSDF interventions reduced the amount of *Firmicutes* and increased the amount of *Bacteroidetes*. In addition, the relative abundance of *Akkermansia* at the genus level was much higher in the FSDF and MIX mice than in the HFD mice (Figure S1). Microbial richness and diversity were estimated using the Chao and Shannon indices (Figure 5B,C). The Chao and Shannon indices were not statistically different among the HFD, FSDF, and MIX groups, and these indices were significantly lower than in the control and FIDF groups, suggesting that the FIDF restored the richness and diversity of the bacterial community which had been reduced by the HFD feeding. PCoA analyses based on Bray–Curtis matrices showed that the bacterial communities of the mice differed under different dietary interventions (Figure 5D). There was an obvious distinction between the HFD group and the control group in the composition of the intestinal flora. Along the PC1 and PC2 axis, the FIDF and FSDF interventions decreased the separation from the control group.

**Figure 6:**
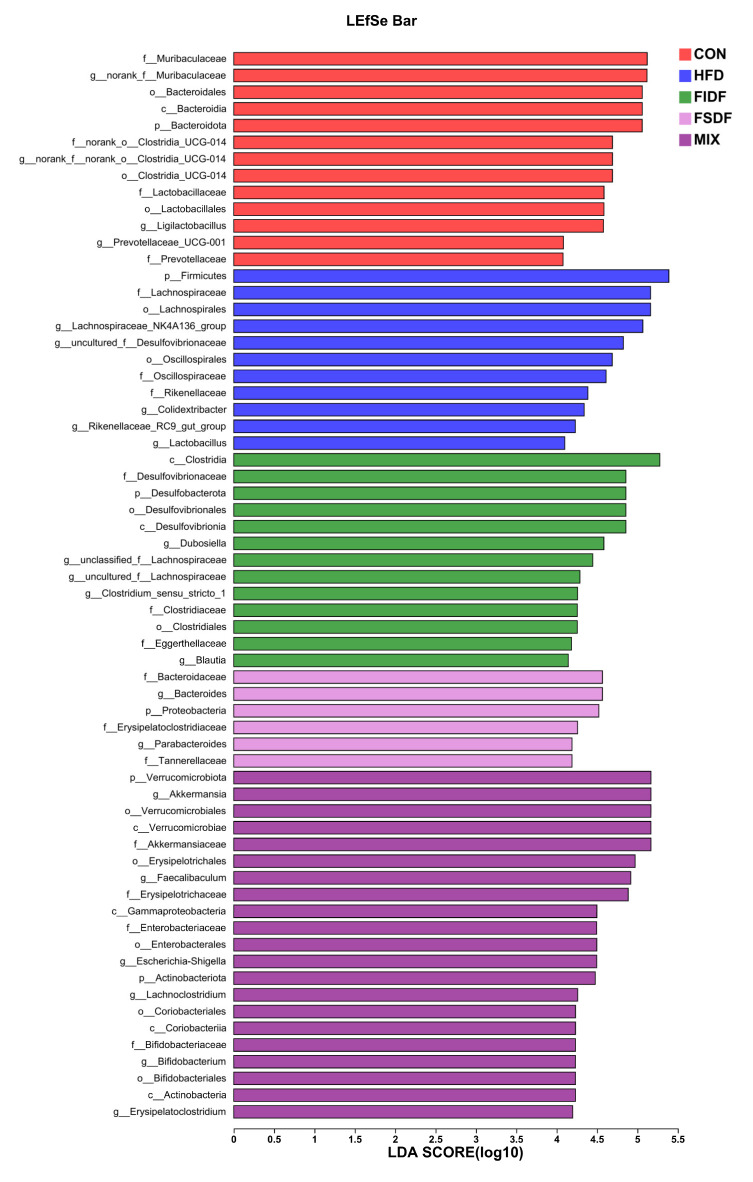
The LEfSe analysis of gut microbiota (LDA scores > 4.0). The characteristic gut microbiota of the different groups are showed in the figure. CON, control group; HFD, high-fat diet group; FIDF, group supplemented with FIDF alone; FSDF, group supplemented with FSDF alone; MIX, group supplemented with FIDF and FSDF together.

Next, we used a LEfSe analysis (LDA > 4) to identify any significant changes in bacterial taxa caused by the different treatments. As is shown in Figure 6, 64 bacterial taxa were found to be key nodes whose abundances were considerably affected by the HFD feeding as well as the FIDF and FSDF interventions. The bacteria *p_Firmicutes*, *f_Lachnospiraceae*, *g_Lachnospiraceae_NK4A136_group*, and *f_Oscillospiraceae* were the key microbes for the HFD group. The FIDF group had a high abundance of *c_Clostridia*, *f_Desulfovibrionaceae*, *g_unclassified_f_Lachnospiraceae*, *f_Eggerthellaceae*, *g_Clostridium_sensu_stricto_1*, and *g_Blautia*, while *g_Bacteroidaceae*, *g_Bacteroides*, *p_Proteobacteria*, *f_Erysipelatoclostridiaceae*, *g_Parabacteroides*, and *f_Tannerellaceae* were more enriched in the FSDF group. The MIX group was distinguished by *g_Akkermansia*, *o_Verrucomicrobiales*, *f_Akkermansiaceae*, *g_Faecalibaculum*, *f_Enterobacteriaceae*, and *g_Bifidobacterium*.

### 3.6. Effects of FIDF and FSDF on SCFA Production

**Figure 7:**
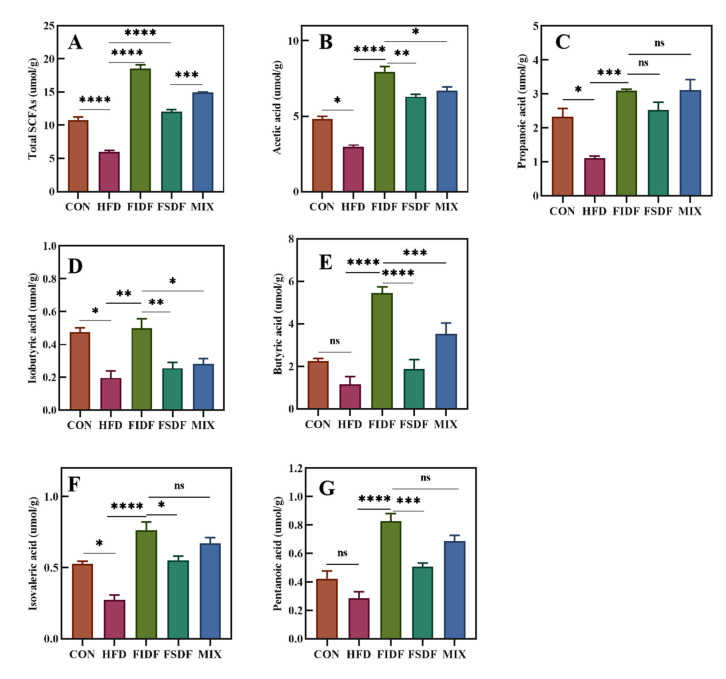
Effects of FIDF and FSDF on SCFA production. Concentrations of (**A**) total SCFAs, (**B**) acetic acid, (**C**) propanoic acid, (**D**) isobutyric acid, (**E**) butyric acid, (**F**) isovaleric acid, and (**G**) pentanoic acid. CON, control group; HFD, high-fat diet group; FIDF, group supplemented with FIDF alone; FSDF, group supplemented with FSDF alone; MIX, group supplemented with FIDF and FSDF together. Data are expressed as mean ± SEM (*n* = 6). * *p* < 0.05; ** *p* < 0.01; *** *p* < 0.0001; **** *p* < 0.0001; ns, not significant.

SCFAs are the main bacterial metabolite produced by specific anaerobic gut bacteria after the fermentation of dietary fibers. The HFD-fed mice had the lowest levels of all six SCFAs (Figure 7). The FIDF and FSDF interventions significantly enhanced the production of all six SCFAs in the cecum of the treated mice compared with the HFD mice. This promotion effect of the FIDF intervention was stronger than that of the FSDF intervention. As is shown in Figure 7A, the FIDF mice had the highest levels of total SCFAs, followed by the MIX mice, the FSDF mice, the control mice, and the HFD mice.

## 4. Discussion

In the present study, two types of dietary fibers with different water solubility (FIDF and FSDF) were extracted from flaxseed cake using the enzymatic hydrolysis method. We then compared their anti-obesity effects and explored the underlying mechanisms.

The compositions and structures of dietary fibers are two important factors determining their health properties [26,27]. Arabinose monosaccharides were present in both the FIDF and FSDF samples. Dietary arabinose has been proven to delay sucrose digestion and attenuate subsequent glucose absorption in the human body [28]. Arabinose acts through selectively inhibiting sucrase activity [29]. Previous studies have shown that polysaccharides extracted from *Morchella esculenta*, comprising glucose, arabinose, mannose, galactose, rhamnose, and so on, attenuate HFD-induced obesity and improve gut microbiota dysregulation [30]. A kind of polysaccharide containing glucose, galactose, xylose, arabinose, and glucuronic acid was proven to enhance the intestinal barrier and modulate the host immune response [31]. Additionally, the galactose, arabinose, and rhamnose content in monosaccharides correlated positively with antioxidant activity [32]. The porous surface of the FIDF and the loose network structure of the FSDF observed during our SEM analyses might increase their adsorption ability and binding capacity. Our findings further support their lipid metabolism regulation ability.

FSDF intake ameliorated serum lipid profiles, reducing the TC, TG, and LDL-c levels in the FSDF-treated mice compared with those of the HFD mice. Combined FSDF and FIDF intake led to a similar effect on the serum lipid profile. FIDF intake produced no effect, however. Our results suggest that only soluble dietary fibers from flaxseed cake can affect serum lipids positively. The TBA levels of the mice in both the FSDF and MIX groups exhibited a distinct rise. This result was consistent with previous findings [33,34]. The increased TBA levels might be related to the significant increase in the amount of *Bacteroides*, which can secrete bile acid metabolizing enzymes and stimulate the production of bile acids [35]. As the largest solid organ in the body, the liver is an important place for fat metabolism and maintains a balance between lipid input (lipid uptake and lipogenesis) and output (lipid export and oxidation) [36,37]. The long-term intake of a high-fat diet could break this balance and lead to abnormal lipid accumulation in the liver and liver steatosis [38]. Based on the results of HE staining images of hepatic cell histomorphology, our findings revealed that the FIDF and FSDF interventions significantly reduced lipid accumulation in the livers of obese mice. The FIDF and FSDF interventions also reduced liver weight and reduced liver TC and TG levels. Despite the fact that the FIDF intervention did not reduce the body weight or the liver weight, it exhibited a greater ability to improve the lipid metabolism in the liver. Studies have shown that, compared with soluble dietary fiber, insoluble dietary fibers have a better ability to improve the obese phenotype in obese mice, e.g., they can reduce weight gain and reduce hepatic triacylglycerol content after 45 weeks of long-term feeding [39]. Our intervention period was five weeks, and it is assumed that the manifestation of the FIDF’s health benefits might require a more prolonged intervention. It will be necessary to conduct longer FIDF and FSDF interventions in obese mice to further verify the anti-obesity effects of FIDF and explore the underlying mechanisms. Our results have demonstrated that the FIDF and FSDF played different roles in the regulation of lipid metabolism. The FSDF was comparatively more effective in lowering body weight and regulating plasma lipid metabolism, while the FIDF exhibited a better ability to alleviate liver lipid accumulation. Due to multifaceted mechanisms in protecting against obesity, a combined FIDF and FSDF treatment might be more effective against obesity than treatment with either dietary fiber alone.

Our results proved that the alleviation of obesity in obese mice was related to increased basal metabolism. The energy intake of dietary fiber-treated and untreated obese mice was roughly the same, and the FIDF and FSDF intake increased the energy consumption, thus offsetting the energy imbalance induced by a high-fat diet. Several mechanisms may explain the DF-induced alleviation of obesity, such as increased energy expenditure, enhanced ambulatory activity, and increased RER level, which indicates increased fat oxidation. The physical activity of the three DF-treated groups at night did not differ from that of the control group. Moreover, the HFD mice showed decreased physical activity, and this could not be due to their high body weight since the FIDF mice had similar body weights.

Dysregulation of the gut microbiota has emerged as an important factor driving obesity progression [40,41]. Gut microbiota play important roles in food digestion and energy absorption in the human body. Our study found that continuous exposure to a high-fat diet changed the intestinal microbial composition of mice significantly, mainly represented by a decrease in the relative abundance of *Bacteroidetes*, an increase in the relative abundance of *Firmicutes*, and the generation of harmful bacteria (e.g., *Desulfovibrionaceae* and *Erysipelotrichaceae*). These two harmful bacteria have previously been reported to be enriched in diet-induced obese mice [42,43]. The intervention with FSDF alone or with FIDF and FSDF together reversed the above changes induced by the HFD. Further, the intervention with the FIDF alone helped restore the richness and diversity of the gut microbiota and reversed the HFD-induced changes slightly by decreasing the *Firmicutes*/*Bacteroidetes* ratio and increasing the quantity of beneficial bacteria, such as *g_Acetatifactor* and butyrate-producing bacterium *g_Clostridium_sensu_stricto* and *g_Blautia*. This increase in the number of SCFA-producing bacteria may have led to the enhancement of SCFA production that we observed in the FIDF mice.

Compared with the HFD group, the group treated with FIDF and FSDF together exhibited an enriched abundance of *g_Akkermansia*, *norank_f_Muribaculaceae*, and butyrate-producing bacterium *g_Bifidobacterium*. *Akkermansia* had been proven to be negatively associated with obesity and its attendant diseases [44], and it can improve a damaged intestinal barrier by enhancing the monolayer integrity of intestinal cells [45]. Our results showed that the intake of FIDF and FSDF together could significantly reduce the ratio of *Firmicutes*/*Bacteroidetes*, promote the relative abundance of probiotics in the gut, and reduce the number of opportunistic pathogens. Moreover, the intervention with FSDF alone also promoted the abundance of *g_Akkermansia* and *norank_f_Muribaculaceae*. Our findings suggest that FIDF and FSDF may mitigate diet-induced obesity partly through modulating intestinal microbiota. In addition, compared with FSDF or FIDF supplementation alone, the intervention with FIDF and FSDF together resulted in relatively lower liver TG levels, higher RER levels, and higher abundance of *g_Akkermansia* and *g_Bifidobacterium*. *g_Akkermansia* and *g_Bifidobacterium* are two of the main beneficial bacteria which are negatively associated with obesity. These results indicate that FIDF and FSDF might have a synergistic effect on obesity treatment, but further experiments are needed to make this clear. Understanding how dietary fibers with different water solubility impact the microbiome, and in turn the lipid metabolism and energy metabolism, is critical to provide guidance on how people might effectively take dietary fiber as a daily dietary supplement. Previous studies have shown that some soluble and insoluble dietary fibers (such as oligofructose and arabinoxylan) could reduce people’s body weight and improve their lipid metabolism. However, we still do not know the synergistic effects of soluble and insoluble dietary fibers on human body weight control [46,47,48]. Our results showed that both the FIDF and the FSDF were indispensable, and that the combined use of them could have a more positive effect on health. In the future, the synergistic effects of FIDF and FSDF on human obesity treatment should be investigated. This will provide valuable recommendations for daily dietary fiber intake and improve human health significantly.

## 5. Conclusions

In our study, we compared the health benefits of FIDF and FSDF extracted from flaxseed cake; specifically, we investigated whether they could reduce obesity and fat accumulation in HFD mice. Our results suggest that FIDF and FSDF may alleviate obesity by increasing basal metabolism, regulating intestinal flora, and promoting SCFA production. Our most important finding was that FIDF and FSDF played different roles in the regulation of the development of obesity. The FSDF was comparatively more potent in lowering body weight, regulating serum lipid metabolism, and improving energy balance, while the FIDF was more effective in reducing liver lipid accumulation and promoting SCFA production. The interventions with FSDF alone or with FIDF and FSDF together showed a strong positive effect on gut microbiota dysbiosis. FIDF and FSDF might have a synergistic effect on obesity treatment, and further detailed studies are required to explore this. Our findings demonstrated the anti-obesity potential of FIDF and FSDF obtained from flaxseed cake as novel dietary fiber resources.

## Figures and Tables

**Table 1:** The monosaccharide compositions (%) of FIDF and FSDF extracted from flaxseed cake. Data are expressed as mean ± SEM from three independent experiments.

Monosaccharide (%)	FIDF	FSDF
Fucose	0.36 ± 0.12	ND
Rhamnose	5.62 ± 0.77	9.91 ± 1.59
Arabinose	8.27 ± 1.06	8.33 ± 0.9
Galactose	5.87 ± 1.47	12.08 ± 1.35
Glucose	37.26 ± 2.35	15.06 ± 2.08
Xylose	16.01 ± 1.16	31.91 ± 1.06
Galacturonic acid	0.45 ± 0.18	3.73 ± 0.67

## Data Availability

The data presented in this study are available within the article.

## Supplementary Materials

The following supporting information can be downloaded at: https://www.mdpi.com/article/10.3390/nu15071718/s1, Table S1: The compositions and heat ratio of the experimental diets; Table S2: Effects of FIDF and FSDF administration on obesity-related parameters; Figure S1: Effects of FIDF and FSDF administration on intestinal flora composition at the genus level.
