# Supplementary material for: Anti-Obesity Effects of Dietary Fibers Extracted from Flaxseed Cake in Diet-Induced Obese Mice
Source: Nutrients. 2023 Mar 31;15(7):1718. doi: 10.3390/nu15071718 (PMC10097256; doi:10.3390/nu15071718)
Supplement: Supplementary file 1 [file nutrients-15-01718-s001.zip › nutrients-2286489-supplementary.pdf]

Table S1. The compositions and heat ratio of the experimental diets.

Ingredient	Diets Compositions (g/100g)				
	**CON**	**HFD**	**FIDF**	**FSDF**	**FIDF+FSDF**
Casin	18.96	25.85	25.85	25.85	25.85
L-Cystine	0.28	0.39	0.39	0.39	0.39
Corn Starch	47.98	0.00	0.00	0.00	0.00
Maltodextrin	11.85	16.15	16.15	16.15	16.15
Sucrose	6.52	8.89	8.89	8.89	8.89
Cellulose	4.74	6.46	0.00	0.00	0.00
FIDF	0.00	0.00	6.46	0.00	3.23
FSDF	0.00	0.00	0.00	6.46	3.23
Soybean Oil	2.37	3.23	3.23	3.23	3.23
Lard	1.90	31.66	31.66	31.66	31.66
Mineral Mix S10026	0.95	1.29	1.29	1.29	1.29
Dicalcium Phosphate	1.23	1.68	1.68	1.68	1.68
Calcium Carbonate	0.52	0.71	0.71	0.71	0.71
Potassium Citrate, 1 H_2O	1.56	2.13	2.13	2.13	2.13
Vitamin Mix V10001	0.95	1.29	1.29	1.29	1.29
Choline Bitartrate	0.19	0.26	0.26	0.26	0.26
	Diets Heat Ratio (%)				
	CON	HFD	FIDF	FSDF	FIDF+FSDF
Protein	20	20	20	20	20
Carbohydrate	70	20	20	20	20
Fat	10	60	60	60	60

CON diet adopted the XTCON50J formulation with energy density of 3.85 kcal/g; HFD diet adopted the XTHF60 formulation with energy density of 5.24 kcal/g; FIDF, FSDF and FIDF+FSDF diet were mainly based upon the XTHF60 formulation with energy density of 5.24 kcal/g. CON, control group; HFD, high-fat diet group; FIDF, group supplemented with FIDF alone; FSDF, group supplemented with FSDF alone; MIX, group supplemented with FIDF and FSDF together.

Table S2. Effects of FIDF and FSDF administration on obesity-related parameters.

Variables	CON	HFD	FIDF	FSDF	MIX
Final body weight (g)	27.13 ± 0.54 ****	41.97 ± 4.31	42.57 ± 3.57 ns	33.15 ± 1.36 ****	37.29 ± 3.56 *
Total Energy Intake During Treatment (kcal)	419.66 ± 5.35 ***	557.80 ± 0.94	541.47 ± 10.69 ns	531.16 ± 5.92 ns	548.73 ± 10.14 ns
Energy Efficiency During Treatment (g/kcal)	0.0654 ± 0.0008 **	0.0753 ± 0.0001	0.0786 ± 0.0002 ns	0.0624 ± 0.0007 **	0.0679 ± 0.0001 *
UA (umol/L)	114.65 ± 22.49 ns	118.8 ± 19.61	100.95 ± 8.65 ns	95.42 ± 8.28 ns	97.35 ± 9.82 ns
ALT (U/L)	480.33 ± 32.75 ***	561.32 ± 23.19	560.53 ± 31.36 ns	551.80 ± 28.71 ns	545.23 ± 24.22 ns
AST (U/L)	191.32 ± 17.33 ns	225.38 ± 29.84	206.583 ± 21.73 ns	174.22 ± 30.36 **	174.68 ± 17.82 **

CON, control group; HFD, high-fat diet group; FIDF, group supplemented with FIDF alone; FSDF, group supplemented with FSDF alone; MIX, group supplemented with FIDF and FSDF together. Data are expressed as mean ± SEM ($n = 6$). * $p < 0.05$, ** $p < 0.01$, *** $p < 0.0001$, **** $p < 0.0001$, ns, not significant; compared with the HFD mice.

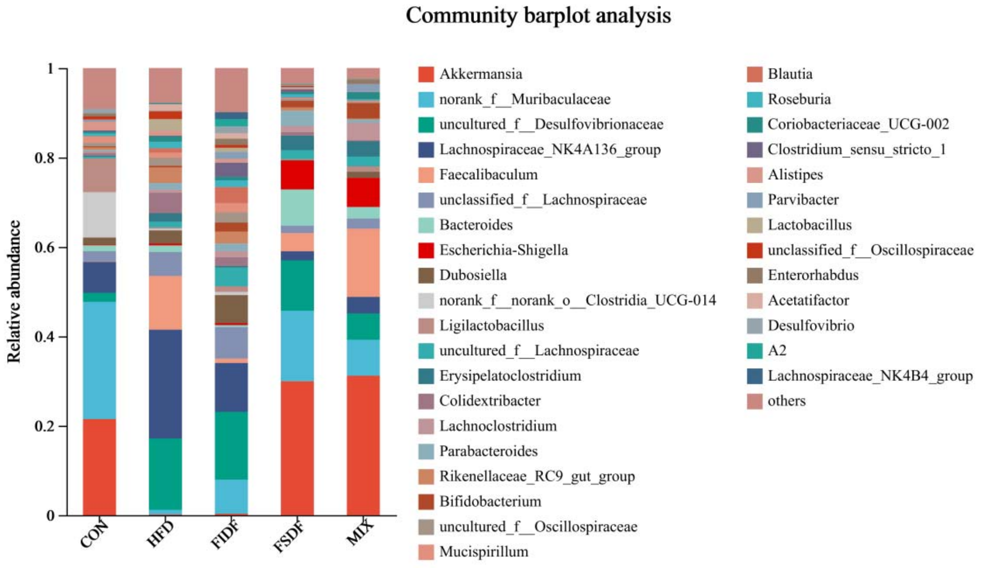

Figure S1. Effects of FIDF and FSDF administration on intestinal flora composition at the genus level. CON, control group; HFD, high-fat diet group; FIDF, supplementation with FIDF alone group; FSDF, supplementation with FSDF alone group; MIX, supplementation with FIDF and FSDF together group.
